# Ribosome profiling reveals differences in global translational vs transcriptional gene expression changes during early *Candida albicans* biofilm formation

**DOI:** 10.1128/spectrum.02195-24

**Published:** 2025-01-28

**Authors:** Vasanthakrishna Mundodi, Saket Choudhary, Andrew D. Smith, David Kadosh

**Affiliations:** 1 Department of Microbiology, Immunology & Molecular Genetics, University of Texas Health Science Center at San Antonio, San Antonio, Texas, USA; 2 Quantitative and Computational Biology, University of Southern California, Los Angeles, California, USA; University of Debrecen, Debrecen, Hungary

**Keywords:** *Candida albicans*, translatome, biofilms, transcriptome, ribosome profiling

## Abstract

*Candida albicans,* a major human fungal pathogen, can form biofilms on a variety of inert and biological surfaces. *C. albicans* biofilms allow for immune evasion, are highly resistant to antifungal therapies, and represent a significant complication for a wide variety of immunocompromised patients in clinical settings. While transcriptional regulators and global transcriptional profiles of *C. albicans* biofilm formation have been well-characterized, much less is known about translational regulation of this important *C. albicans* virulence property. Here, using ribosome profiling, we define the first global translational profile of genes that are expressed during early biofilm development in a human fungal pathogen, *C. albicans*. We show that *C. albicans* biofilm formation involves altered translational regulation of genes and gene classes associated with protein synthesis, pathogenesis, transport, plasma membrane, polarized growth, cell cycle, secretion, and signal transduction. Interestingly, while similar, but not identical, classes of genes showed transcriptional alterations during early *C. albicans* biofilm development, we observed very little overlap between specific genes that are upregulated or downregulated at the translational vs transcriptional levels. Our results suggest that distinct translational mechanisms play an important role in regulating early biofilm development of a major human fungal pathogen. These mechanisms, in turn, could serve as potential targets for novel antifungal strategies.

**IMPORTANCE:**

The major human fungal pathogen *Candida albicans* is known to form biofilms or complex aggregated microbial communities encased in an extracellular matrix. These biofilms allow *C. albicans* to escape detection by the immune system as well as resist a variety of antifungal drugs. In this study, we define the first global profile of genes that show altered translation during *C. albicans* biofilm formation. These genes are involved in a variety of key cellular processes, including polarized growth, pathogenesis, transport, protein synthesis, cell cycle, plasma membrane, signal transduction, and secretion. Interestingly, while similar classes of genes are induced at both the transcriptional and translational levels during early *C. albicans* biofilm formation, we observed very little overlap among specific genes with altered transcription and translation. Our results suggest that *C. albicans* biofilm formation is controlled by distinct translational mechanisms, which could potentially be targeted by novel antifungal drugs.

## INTRODUCTION

Candidiasis represents the fourth leading cause of hospital-acquired bloodstream infections in the United States (1–3). A variety of immunocompromised individuals, such as AIDS patients and cancer patients on chemotherapy, are susceptible to infection (4–8). In addition, at least 70% of all women will experience a vaginal candidiasis infection in their lifetime (1). Approximately $1 billion per year is spent on the treatment of hospital-acquired *Candida* infections (9, 10). The majority of these infections can be attributed to *Candida albicans*, the most frequently isolated human fungal pathogen (11).

A key pathogenicity trait of *C. albicans* is the ability to form biofilms or complex highly structured cellular microbial communities that are encased by an extracellular matrix (12, 13). *C. albicans* biofilms can form on a variety of host surfaces, such as the oral and vaginal mucosa, parenchymal organs, as well as epithelial cell linings. In addition, biofilms can form on a variety of implanted medical devices, including catheters, dentures, pacemakers, and joint prostheses (13–16). Because these biofilms can evade host immune defenses and are highly resistant to current antifungal therapies, they pose a significant complication for both individual patients and clinicians (17–21).

*C. albicans* biofilm development occurs in four distinct phases (13, 16). During the adherence phase, yeast cells adhere to a solid surface to form a basal layer, which anchors the biofilm. The initiation phase involves subsequent growth of biofilm cells in yeast, pseudohyphal and hyphal forms. During the maturation phase, a complex, structured biofilm forms that includes extended hyphal filaments and is surrounded by an extracellular matrix. In the dispersion phase, individual cells from *C. albicans* biofilms can detach and travel through the bloodstream to establish additional biofilms and secondary infections (13, 16).

Several previous *in vitro* whole-genome transcriptional profiling studies have shown that *C. albicans* biofilms are under widespread transcriptional control (22–27). During early-stage biofilm development (6 hours, following the adhesion phase), genes important for amino acid metabolism, intracellular transport, cell wall synthesis, stress responses, sulfur metabolism/assimilation, glycolysis, DNA synthesis, chromatin assembly, phosphate metabolism, and lipid biosynthesis are induced (23, 27). Adhesins were found to be upregulated at both early and late stages of biofilm formation, whereas genes associated with metabolism were generally downregulated in mature biofilms (26). Similar, but not identical, transcriptional patterns were observed in *in vivo* biofilms formed on a rat catheter (24). A limited proteomics analysis has also shown that proteins associated with similar, but not identical, gene classes are induced upon biofilm formation (28). Approximately 50 transcription factors are known to regulate *C. albicans* biofilm development (13). Six of these factors, termed “master regulators,” control each other and form a complex regulatory network that affects the expression of multiple target genes (25). A number of these genes play important roles in matrix production, adhesion, drug resistance, and hyphal formation (13, 25). Post-translational control of *C. albicans* biofilm formation has also been well-documented. Both surface contact-dependent kinase mutants, such as *mkc1*Δ/Δ, as well as cell wall-defective kinase mutants, such as *cbk1*Δ/Δ, are defective for biofilm formation (29–31). In addition, several alcohol dehydrogenases are also important for this process (32–35).

In contrast to transcriptional and post-translational mechanisms, very little is known about translational mechanisms that control *C. albicans* biofilm development. However, the Ccr4-Pop2 mRNA deadenylase, a key regulator of mRNA stability and translation, has been shown to control cell wall integrity and biofilm formation (36). In addition, previous studies have demonstrated that expression of both *UME6* and *EFG1*, important transcriptional regulators of *C. albicans* filamentation and biofilm development, is controlled by 5’ untranslated region (UTR)-mediated translational efficiency mechanisms (37, 38). Finally, a previous RNA-seq analysis has shown that, similar to *UME6*, several known regulators of *C. albicans* biofilm formation, including members of the α-agglutinin-like (ALS) family of adhesins, possess unusually long 5’ UTRs and could potentially be under translational control (39).

In this study, to gain a better understanding of global translational mechanisms that control *C. albicans* biofilm formation, we used a more recently developed and powerful approach, ribosome profiling (40). This technique provides genome-wide identification of all sequences that are bound to ribosomes and allows us to determine several key aspects of translation, including translational efficiency as well as locations of actively translating genes. A major advantage over transcriptional profiling experiments is that ribosome profiling provides a more accurate proxy for actual protein levels (41, 42). Here, using this approach, we demonstrate that *C. albicans* biofilm formation is under widespread translational control and identify both genes and gene classes that show altered translational efficiency during early *C. albicans* biofilm development. Importantly, we also observe distinct differences between transcriptional and translational gene expression changes associated with *C. albicans* biofilm formation, highlighting the importance of translational control. New information gained from this study about global translational regulation of *C. albicans* biofilm formation may eventually be used to inform the development of novel antifungals that target this important pathogenicity trait.

## RESULTS

### Ribosome profiling of *C. albicans* cells during early biofilm development

**Fig 1:**
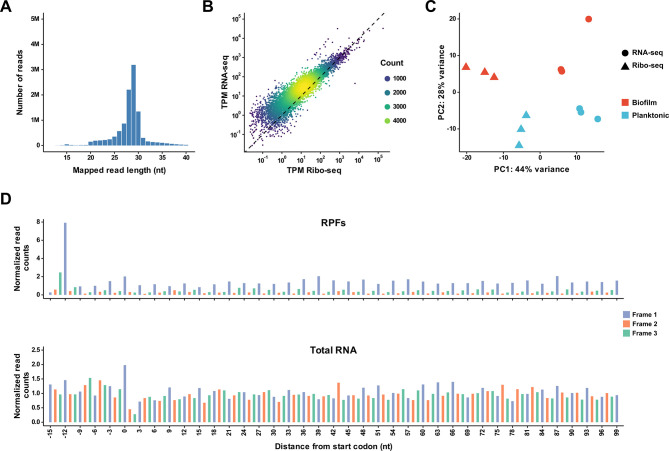
Ribosome profiling data quality analysis. (**A**) Example read length distribution plot of ribosome protected fragments (RPFs) for a sample grown under planktonic conditions. (**B**) Scatter plot demonstrating a general correlation between Ribo-seq and RNA-seq read counts for the sample in part (**A**). (**C**) Principal component analysis (PCA) for Ribo-seq and RNA-seq samples grown under biofilm and planktonic conditions (all biological replicates are shown). (**D**) Example metagene reading frame enrichment plots, generated with a 29 nt. read length, for both RPF and total RNA for the sample in part (**A**). Normalized read counts at each position relative to the start codon (position 0) for genes are shown. TPM = transcripts per million; nt = nucleotide.

A *C. albicans* wild-type strain was grown in RPMI-1640 medium at 37°C under biofilm or planktonic control conditions. Separate aliquots of cells were harvested at the 6-hour time point for Ribo-seq and RNA-seq analysis. This time point was selected because previous transcriptional profiling studies have shown that large changes in gene expression occur during early biofilm development (23, 27). Ribosome profiling was carried out using an optimized protocol for *C. albicans* that we have described previously (43, 44). Initial quality control analyses indicated that the majority of ribosome-protected fragment (RPF) reads were in the 27–30 nucleotide (nt.) range (Fig. 1A), which is consistent with RPF read lengths that we have previously observed in *C. albicans* and that have been documented for other organisms (43–45). We also observed a general genome-wide correlation between RNA-seq and Ribo-seq read counts, as expected (Fig. 1B). In addition, a principal component analysis (PCA) demonstrated distinct groupings for all three biological replicates of RNA-seq and Ribo-seq samples from cells grown under both biofilm and planktonic conditions (Fig. 1C). The consistency of our data was confirmed by a pairwise analysis of all combinations of Ribo-seq (Fig. S1) and RNA-seq (Fig. S2) sample biological replicates. Finally, a 3-nucleotide periodicity metagene plot demonstrated clear periodicity in RPF samples and a lack of periodicity in total RNA samples, as expected (Fig. 1D). The P-site offset was indicated by a large peak in the RPF sample at the −12 nt. position relative to the start codon. As an additional indication of the significance of periodicity, all RPF samples showed a phase score (44, 46) >0.41. These findings validate the quality of the ribosome profiling data set that we used in this study to identify global translational alterations in *C. albicans* gene expression in biofilm vs planktonic cells.

### Global translational profile of genes expressed during *C. albicans* early biofilm development

**Fig 2:**
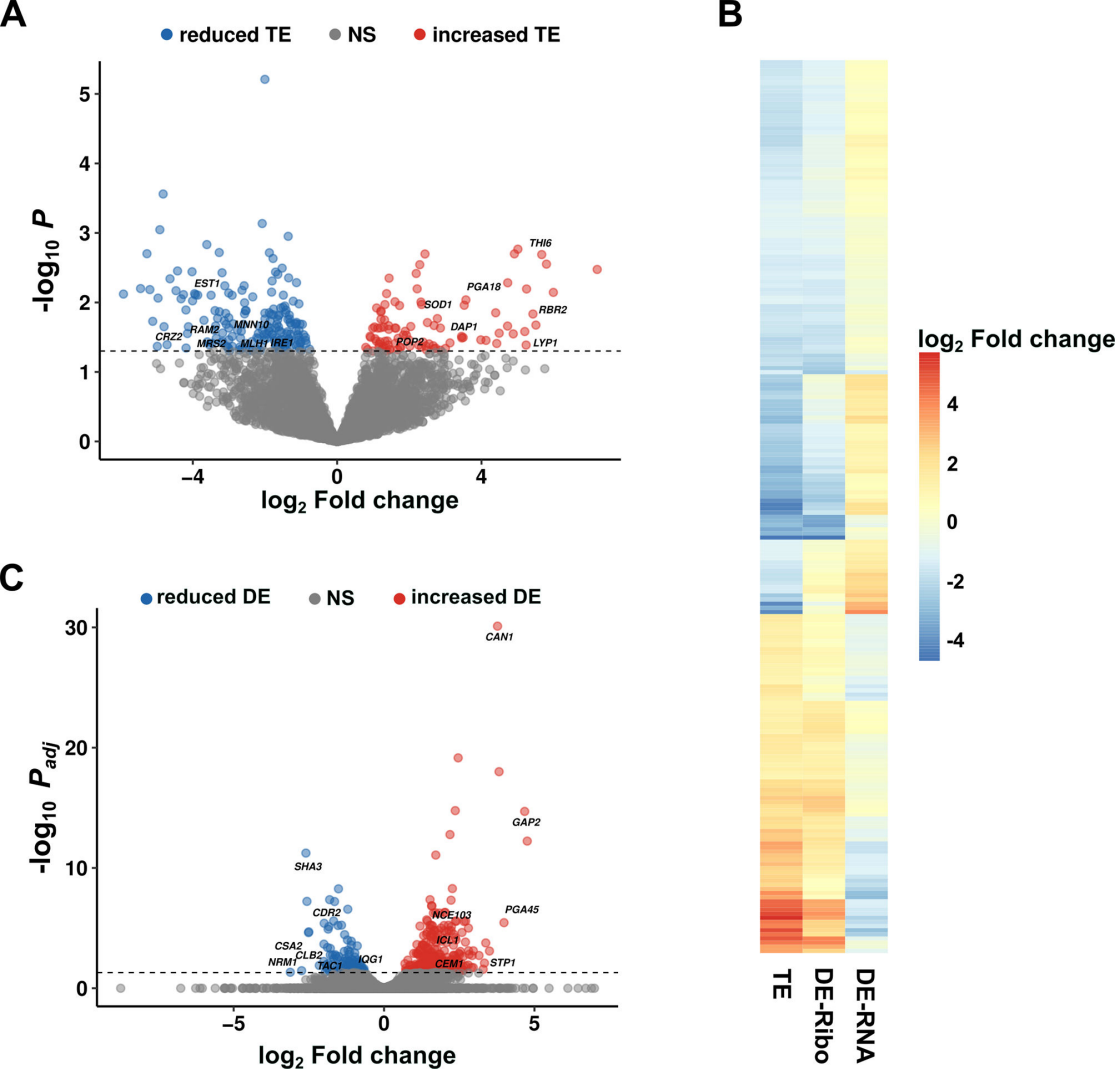
Identification of *C. albicans* genes showing altered translational efficiency (TE) and RNA differential expression (DE) under biofilm vs planktonic growth conditions. (**A**) Volcano plot showing genes with alterations in TE when cells were grown under biofilm vs planktonic conditions. Horizontal dashed line indicates a cutoff of *p* = 0.05. (**B**) Heat map showing genes with altered TE when cells were grown under biofilm vs planktonic conditions as defined in part (**A**) and Table 1. Corresponding differential expression (DE) values for both Ribo-seq (DE-Ribo) and RNA-seq (DE-RNA) when cells were grown under biofilm vs planktonic conditions are shown. (**C**) Volcano plot showing genes with altered RNA DE when cells were grown under biofilm vs planktonic conditions. Horizontal dashed line indicates *p*_adj_ = 0.05 cutoff; NS = nonsignificant.

**Fig 3:**
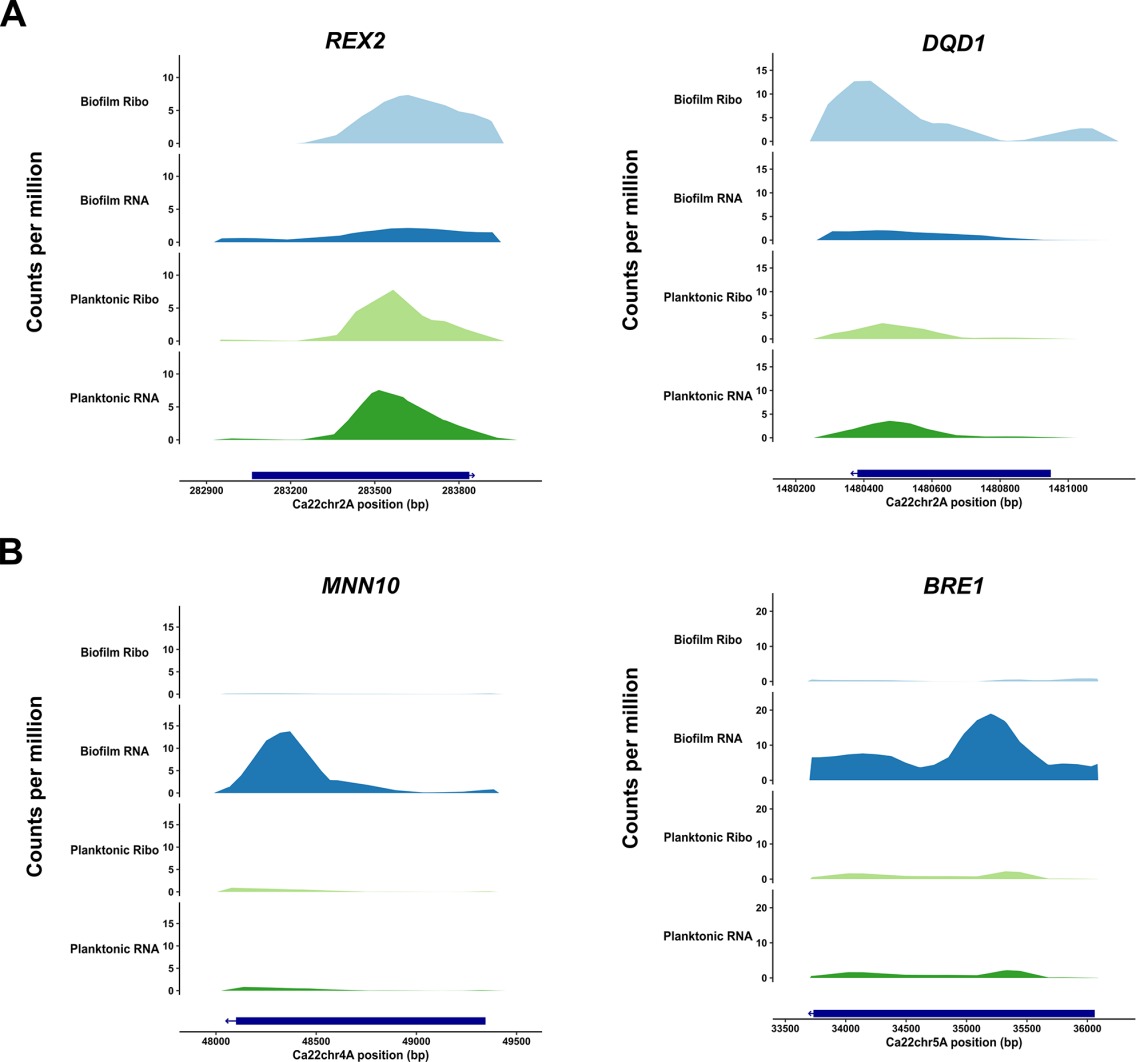
Read coverage plot examples for selected *C. albicans* genes showing differential translational efficiency (TE) when cells were grown under biofilm vs planktonic conditions. Normalized RNA-seq and Ribo-seq average read coverage across all replicates is shown for *REX2* and *DQD1* (**A**), which show increased TE, as well as *MNN10* and *BRE1* (**B**), which show reduced TE.

By comparing Ribo-seq and RNA-seq reads, we initially determined the translational efficiency (TE) of each *C. albicans* gene in biofilm vs planktonic cells. Please note that due to the inherent variability in ribosome profiling data (46, 47), it was necessary to use non-adjusted *P* values to determine genes showing the greatest differences in translational efficiency. We observed that 82 genes showed increased TE and 134 genes showed reduced TE (Fig. 2A; Table 1; Data set S1). A significant number of these genes showed large (≥ 4 fold) alterations in translation (Table 1; Data set S1). Approximately two-thirds of genes with increased TE showed increased differential expression of Ribo-seq reads, and one-third showed reduced differential expression of RNA-seq reads (Fig. 2B; Data set S1). Several examples of Ribo-seq vs RNA-seq read coverage under biofilm and planktonic growth conditions for genes with reduced or increased TE are shown in Fig. 3 and Fig. S3.

**TABLE 1:** Number of genes showing significantly altered translational efficiency and RNA differential expression during biofilm formation of *C. albicans*

	≥ 2-fold	≥ 4-fold	≥ 8-fold
# of genes showing increased TE^*a*^	82	41	19
# of genes showing reduced TE^*a*^	134	53	20
# of genes showing increased RNA DE^*b*^	305	99	10
# of genes showing reduced RNA DE^*b*^	81	8	1

^*a*^ Fold changes are based on mean translational efficiency (TE) values in cells grown in biofilm vs planktonic cells at 37°C, from three independent experiments (n = 3, TPM >1 in at least two replicates, *p* ≤ 0.05).

^*b*^ Fold changes are based on mean RNA differential expression (DE) values in cells grown in biofilm vs planktonic cells at 37°C, from three independent experiments (n = 3, TPM >1 in at least two replicates, *p*_adj_ ≤ 0.05).

**Fig 4:**
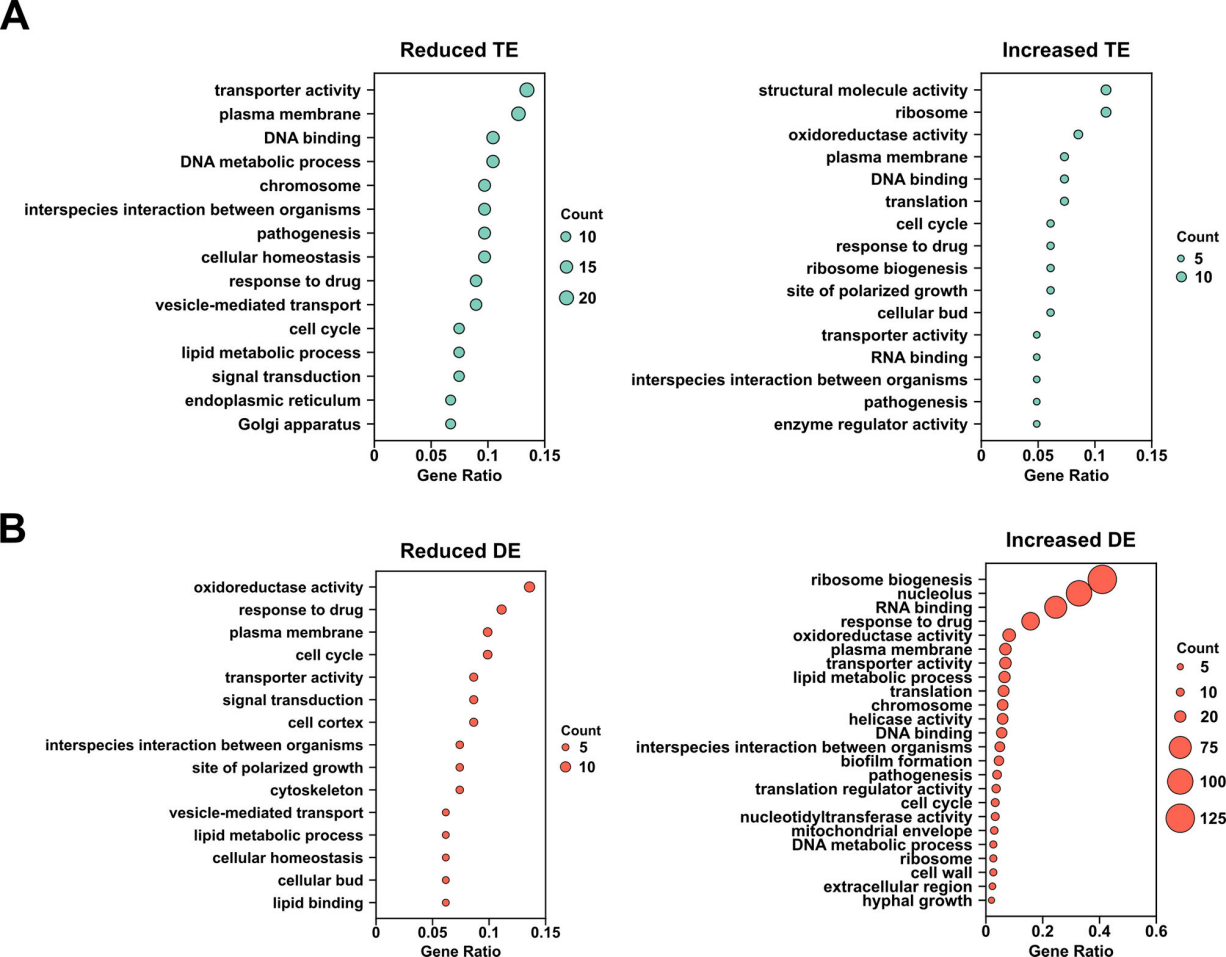
Analysis of gene ontology (GO) for genes showing altered TE and RNA DE under biofilm vs planktonic conditions. (**A**) *C. albicans* GO Slim ontology (*Candida* Genome database, http://www.candidagenome.org/) and clusterProfiler (48) were used to classify genes showing significant changes in TE, as defined in Table 1, by GO terms. (**B**) Genes showing significant changes in RNA DE, as defined in Table 1, were classified by GO terms as described in part (**A**). Count = number of genes within each GO term.

A gene ontology (GO) analysis indicated that gene categories associated with protein synthesis, structural molecules, plasma membrane, transporters, DNA- and RNA-binding activities as well as polarized growth, cell cycle, and pathogenesis are highly represented among the set of genes showing increased TE in biofilm vs planktonic cells (Fig. 4A; Data set S2). Genes encoding cell wall proteins with strong increases in TE included *RBR2*, which is induced in macrophages, as well as the putative GPI-linked proteins *PGA18* and *PGA33* (Table 2; Data set S1). *THI6*, encoding a fungal-specific Spider medium biofilm-induced protein important for thiamine biosynthesis was also strongly upregulated at the translational level during early *C. albicans* biofilm formation, highlighting the demand for nutrients. Multiple signaling components, including *PTC8* protein phosphatase and *RDI1* putative rho GDP dissociation inhibitor, both involved in hyphal growth, as well as the *RNA1* putative GTPase-activating protein, showed increased TE (Table 2; Data set S1). Interestingly, *NRG1*, a strong transcriptional repressor of filamentous growth, was also found in this gene set. Finally, genes important for cytokinesis (*CYK3* and *HOF1*), phosphate transport/metabolism (*PHO88* and *PHO85*), DNA damage repair and oxidative stress responses (*DAP1* and *SOD1*) as well as a component of the Ccr4-Pop2 mRNA deadenylase complex (*POP2*) displayed increased translation (Table 2; Data set S1). Interestingly, while several of these genes have previously been shown to be transcriptionally induced in *C. albicans* biofilms formed under different conditions, others are known to be transcriptionally repressed (Table 2), suggesting that translational regulation plays an important role in modulating their protein levels.

**TABLE 2:** Selected genes showing significantly increased translational efficiency (TE) in *C. albican*s biofilm vs planktonic cells

Gene name^*a*^	Ref. #	Description^*a*^	Fold change in TE^*b*^
*RBR2*	orf19.532	Cell wall protein; transcript regulated upon white-opaque switching; macrophage-induced	38.0
*THI6*	orf19.277	Putative thiamin-phosphate pyrophosphorylase, hydroxyethylthiazole kinase; fungal-specific; Spider biofilm induced	32.2
*LYP1*	orf19.651	Putative permease; amphotericin B induced; possibly an essential gene	21.4
*DAP1*	orf19.489	Similar to mammalian membrane-associated progesterone receptors involved in DNA damage response; induced in core stress response; Hog1 regulated	15.8
*PGA18*	orf19.301	Putative GPI-anchored protein; regulated by Nrg1, Tup1; rat catheter biofilm repressed	11.9
*SOD1*	orf19.2770.1	Cytosolic copper- and zinc-containing superoxide dismutase; role in protection from oxidative stress; required for full virulence; induced by human blood; rat catheter, flow model, and Spider biofilm repressed	11.5
*CYK3*	orf19.6242	Essential protein involved in cytokinesis; contains an SH3 domain	10.9
*REX2*	orf19.1466	Putative 3'−5' RNA exonuclease with a predicted role in 3'-end processing of U4 and U5 snRNAs, 5S and 5.8S rRNAs; rat catheter biofilm induced	8.7
*TPT1*	orf19.5432	tRNA 2'-phosphotransferase; enzyme of tRNA splicing	7.3
*DQD1*	orf19.2283	Putative 3-dehydroquinate dehydratase; ketoconazole-repressed; protein abundance downregulated by macrophages; flow model biofilm induced	6.6
*ATP18*	orf19.2066.1	F_1_F_0_ ATP synthase complex subunit	6.3
*POP2*	orf19.5734	Component of the Ccr4-Pop2 mRNA deadenylase	5.6
*PGA33*	orf19.876	Putative GPI-anchored protein of unknown function; Spider biofilm repressed	5.2
*PHO88*	orf19.7327	Protein with a role in phosphate transport; biofilm-regulated expression; amphotericin B repressed	4.6
*HOF1^c^*	orf19.5664	Protein involved in cytokinesis and DNA damage response	4.1
*CTP1*	orf19.5870	Putative citrate transport protein; flucytosine induced; amphotericin B repressed, caspofungin repressed	3.6
*PTC8*	orf19.4698	Predicted type 2C protein phosphatase, Ser-/Thr-specific; required for hyphal growth; transcript induced by stress; flow model biofilm induced; Spider biofilm induced	3.3
*PCL7^c^*	orf19.1089	Putative cyclin-like protein; possible Pho85 cyclin; hyphal repressed	3.2
*RDI1*	orf19.5968	Putative rho GDP dissociation inhibitor; transposon mutation affects filamentous growth; farnesol, filament-induced; regulated by Nrg1 and Tup1	3.1
*RNA1*	orf19.1649	Putative GTPase-activating protein; Spider biofilm repressed	3.0
*RPS30*	orf19.4375.1	Putative 40S ribosomal protein S30; rat catheter biofilm repressed	2.7
*PHO85*	orf19.6846	Functional homolog of *S. cerevisiae* Pho85, a cyclin-dependent kinase that regulates transcription of *PHO* genes involved in phosphate metabolism	2.5
*NRG1*	orf19.7150	Transcription factor/repressor; regulates chlamydospore formation/hyphal gene induction/virulence and rescue/stress response genes; flow model biofilm induced; Spider biofilm repressed	2.3
*NDT80*	orf19.2119	Ortholog of *S. cerevisiae* Ndt80; activator of *CDR1* induction by antifungal drugs; required for wild-type drug resistance and for Spider biofilm formation	2.1

^*a*^ Gene names and descriptions based on *Candida* Genome Database annotation (http://www.candidagenome.org).

^*b*^ indicates mean fold-change in TE (n = 3, TPM >1 in at least 2 replicates, *p* ≤ 0.05) in cells grown in biofilm vs planktonic RPMI 1640 cultures at 37°C for 6 hours.

^*c*^ indicates that RNA DE is reduced for this gene, as defined in Table 1.

Based on GO analysis, gene classes showing reduced TE during early *C. albicans* biofilm formation included transporters, plasma membrane proteins, DNA-binding proteins, as well as cell cycle and signal transduction components and several classes associated with secretion (vesicle-mediated transport, Golgi apparatus, and endoplasmic reticulum) (Fig. 4A; Data set S2). DNA-binding proteins showing reduced TE included the transcription factors *BRE1*, associated with filamentous growth, and *CRZ2*, which is important for *C. albicans* adherence (Table 3; Data set S1); interestingly, *CRZ2* is known to be transcriptionally induced in biofilms (25). Signaling components included *RAM2*, a geranylgeranyltransferase known to target the Cdc42 master hyphal growth regulator, *IRE1* kinase, important for cell wall regulation, *HRK1* kinase, a putative regulator of cellular ion homeostasis, *TPK2*, a cAMP-dependent protein kinase that controls filamentous growth, and the *PTP1* phosphotyrosine-specific protein phosphatase (Table 3; Data set S1). In addition, genes associated with magnesium (*MRS2*), urea (*NPR2*), ion (*HOL4*), and potassium (*TRK1*) transport all showed reduced TE in biofilm vs planktonic cells (Table 3; Data set S1). Genes involved in secretion with reduced TE included *SEC10*, involved in exocytosis and Golgi-to-plasma membrane transport, and *PEP1*, a type I transmembrane sorting receptor. Interestingly, two genes associated with proteasome-mediated protein degradation, *RTT101* ubiquitin ligase subunit and *BLM3* proteasome activator, also showed decreased TE (Table 3; Data set S1). *ALS1*, a key adhesin important for virulence, which has previously been shown to be transcriptionally induced during biofilm formation *in vitro* (22, 49), was reduced in TE in biofilm vs planktonic cells. Several other genes with reduced TE were also previously shown to be transcriptionally induced during *C. albicans* biofilm formation under different conditions, including *CRZ2*, *IRE1*, *PTP1*, *HOL4,* and *TRK1* (Table 3; Data set S1). These findings, together with observations from the set of genes showing increased TE, suggest that many important genes associated with *C. albicans* biofilm formation demonstrate complex translational regulation that is not necessarily consistent with transcriptional gene expression patterns.

**TABLE 3:** Selected genes showing significantly reduced translational efficiency (TE) in *C. albicans* biofilm vs planktonic cells

Gene name^*a*^	Ref. #	Description^*a*^	Fold change in TE^*b*^
*CRZ2*	orf19.2356	C2H2 transcription factor; required for yeast cell adherence to silicone substrate; Spider biofilm induced	−24.8
*RAM2*	orf19.4817	Alpha subunit of heterodimeric protein geranylgeranyltransferase type I and farnesyltransferase; lovastatin, fluconazole regulated; Cdc42 substrate; rat catheter biofilm repressed	−9.3
*MRS2*	orf19.2597	Putative magnesium ion transporter, mitochondrial; fungal-specific (no human or murine homolog)	−6.5
*MLH1*	orf19.4162	Putative mismatch repair protein	−6.5
*EST1*	orf19.4045	Telomerase subunit; allosteric activator of catalytic activity, but not required for catalytic activity	−5.9
*MNN10*	orf19.5658	Alpha-1,6-mannosyltransferase involved in biosynthesis and organization of cell wall polysaccharides	−4.7
*IRE1*	orf19.5068	Putative protein kinase; role in cell wall regulation; mutant is hypersensitive to caspofungin; Spider biofilm induced	−4.6
*BRE1*	orf19.976	Putative transcription factor with C3HC4 zinc finger DNA-binding motif; transposon mutation affects filamentous growth	−4.2
*SEC10*	orf19.3086	Ortholog(s) have a role in Golgi-to-plasma membrane transport, cell separation after cytokinesis and exocytosis	−4.0
*PEP1*	orf19.3767	Type I transmembrane sorting receptor for multiple vacuolar hydrolases; cycles between late-Golgi and prevacuolar endosome-like compartments; rat catheter biofilm repressed	−4.0
*RTT101*	orf19.2440	Putative cullin subunit of E3 ubiquitin ligase complex, involved in response to DNA damage	−4.0
*NPR2*	orf19.328	Putative urea transporter; induced during infection of murine kidney, compared to growth *in vitro*; has murine homolog	−3.9
*BLM3*	orf19.2182	Putative proteasome activator; binds core proteasome and stimulates proteasome-mediated protein degradation by inducing gate opening	−3.7
*PTP1*	orf19.6365	Phosphotyrosine-specific protein phosphatase; rat catheter biofilm induced	−3.7
*MON2*	orf19.4939	Peripheral membrane protein; role in endocytosis and vacuole integrity; flow model and rat catheter biofilm repressed	−3.5
*HRK1*	orf19.5408	Putative serine/threonine kinase; predicted role in cellular ion homeostasis; Spider biofilm repressed	−3.4
*LRO1*	orf19.6018	Acyltransferase that catalyzes diacylglycerol esterification of phospholipids; role in lipid storage, triglyceride biosynthesis; flow model biofilm repressed	−3.3
*TPK2*	orf19.2277	cAMP-dependent protein kinase catalytic subunit; involved in regulation of filamentation, phenotypic switching and mating; needed for epithelial cell damage, engulfment and oral virulence in mice	−3.1
*HOL4*	orf19.4546	Putative ion transporter; caspofungin repressed; rat catheter and Spider biofilm induced	−3.1
*GLT1*	orf19.6257	Putative glutamate synthase; rat catheter biofilm repressed	−3.0
*TRK1*	orf19.600	Potassium transporter; mediates K^+^ and Cl^–^ influx; Spider biofilm induced	−3.0
*ALS1^c^*	orf19.5741	Cell-surface adhesin; adhesion, virulence, immunoprotective roles; Spider biofilm induced; flow model biofilm repressed	−2.7

^*a*^ Gene names and descriptions based on *Candida* Genome Database annotation (http://www.candidagenome.org).

^*b*^ indicates mean fold-change in TE (n = 3, TPM >1 in at least two replicates, *p* ≤ 0.05) in cells grown in biofilm vs planktonic RPMI 1640 cultures at 37°C for 6 hours.

^*c*^ indicates that RNA DE is increased for this gene, as defined in Table 1.

Because ribosome profiling experiments require the acquisition of RNA-seq data, we were also able to re-examine transcriptional changes in gene expression during early *C. albicans* biofilm development. Overall, 305 genes showed increased RNA differential expression (DE), and 81 genes showed reduced RNA DE (Fig. 2C; Table 1; Data set S1). About one-third of all induced genes were induced ≥4 fold (Table 1; Data set S1).

GO analysis of the set of genes showing increased RNA DE during early *C. albicans* biofilm formation indicated strong representation of gene classes associated with protein synthesis, plasma membrane/cell wall, lipid metabolic processes, nucleic acid binding activity, as well as biofilm formation, pathogenesis, and hyphal growth (Fig. 4B; Data set S2). Consistent with previous reports (27, 49), a significant number of genes associated with amino acid transport and metabolism were transcriptionally induced, including the *GAP2* broad specificity amino acid permease, *CAN1* and *CAN2* basic amino acid permeases, *RIT1* putative initiator tRNA methionine ribosyltransferase, and *MUP1* putative high-affinity methionine permease (Data set S1). Genes important for fatty acid metabolism included *CEM1*, an acyl carrier protein, *TAZ1*, a putative lyso-phosphatidyl acetyltransferase, and *FAD2*, a delta-12 fatty acid desaturase. Transcriptionally induced adhesins included the *TRY5* transcription factor and two members of the *ALS* gene family (*ALS1* and *ALS4*). *HGT2* and *MAL31*, glucose and high-affinity maltose transporters, respectively, as well as the *ALP1* cystine transporter, were also transcriptionally induced during early *C. albicans* biofilm formation (Data set S1). As previously reported (23), several genes involved in sulfur assimilation/metabolism (*MET3* and *MET14*), in addition to the *NCE103* carbonic anhydrase, were induced. Transcriptionally induced genes associated with protein synthesis included *TIF3*, *RPL7*, *SGD1*, *MAK16*, *RPA34*, *MTG1*, *RRS1*, *RPL15A,* and *NIP1* (Data set S1). We also observed biofilm transcriptional induction of a significant number of nucleolar genes, including *RRP15*, *ENP2*, *DRS1*, *NOC2*, *NOC4*, *NOP1*, *NOP4*, *NOP5*, *NOP8*, *NOP13*, *NOP14,* and *NOP15* (Data set S1).

Based on GO analysis, gene classes associated with oxidoreductase activity, response to drug, cell cycle, cell cortex, transporters, polarized growth, budding, cytoskeleton, cellular homeostasis, and lipid metabolic processes were strongly represented among the set of genes transcriptionally downregulated during early *C. albicans* biofilm formation (Fig. 4B; Data set S2). Transporters included *SHA3*, a putative Ser/Thr kinase associated with glucose transport, *HGT18*, also a putative glucose transporter, the multidrug transporter *CDR2*, *TPO3*, a putative polyamine transporter, and *SFH5*, a putative phosphatidylinositol transporter that had previously been shown to be repressed in rat catheter and Spider biofilms (24, 25) (Data set S1). Cell cycle genes included the transcriptional regulator *NRM1* as well as the *CLB2* B-type mitotic cyclin and *PCL7* putative cyclin-like protein. Genes involved in budding/cytokinesis included *ASE1*, a putative microtubule-associated protein, *HOF1*, a bud neck protein, and *IQG1*, a bud neck actomyosin ring component (Data set S1). We also observed downregulation of genes involved in lipid metabolism (e.g., *LCB4* putative sphingolipid kinase and *PLC1* phosphoinositide-specific phospholipase C), exocytosis (*SEC3*), energy production (*MRF1* putative mitochondrial respiratory protein), and metabolism (*PYC2* putative pyruvate carboxylase) (Data set S1). Interestingly, a gene involved in heme/iron acquisition (*CSA2*) and a transcription factor important for iron acquisition (*IRO1*) were also transcriptionally repressed (Data set S1).

### Differences in genes regulated at the translational vs transcriptional level during early *C. albicans* biofilm formation

**Fig 5:**
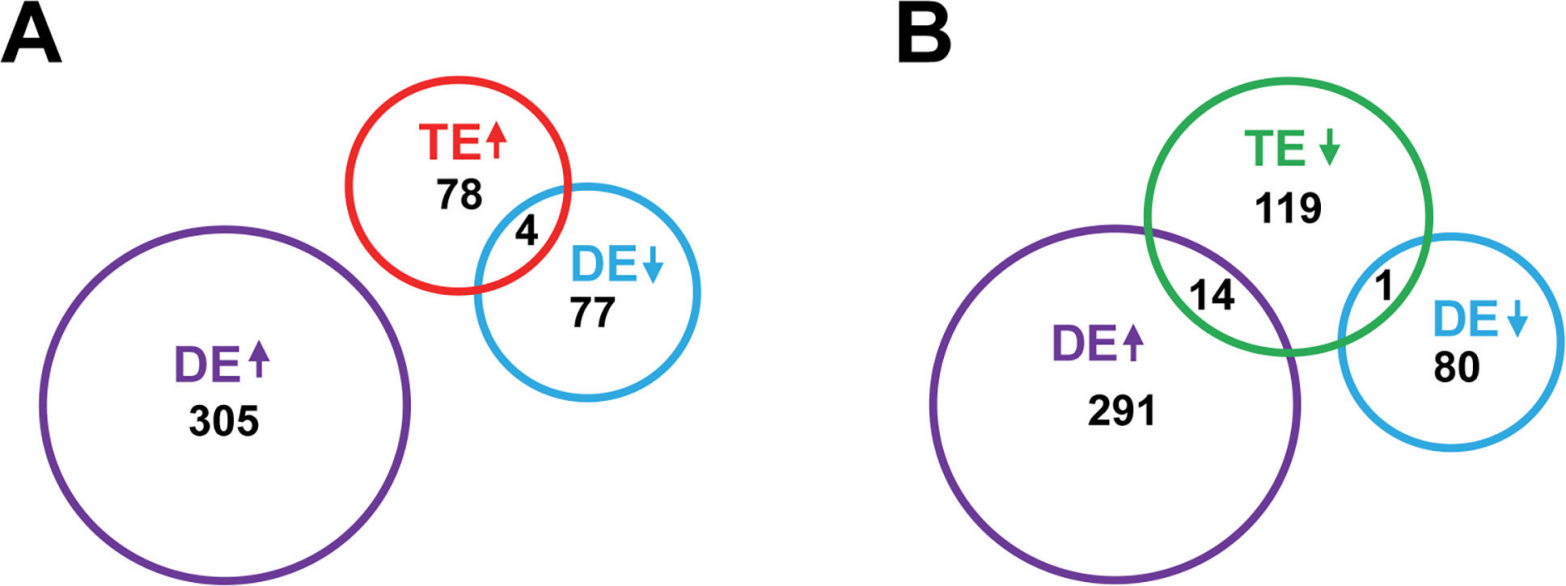
Minimal overlap between genes with altered translational vs transcriptional gene expression during early *C. albicans* biofilm formation. Venn diagram showing overlap between *C. albicans* genes with increased (**A**) or decreased (**B**) translational efficiency (TE) and those with increased or decreased RNA differential expression (DE) under biofilm vs planktonic conditions. Genes with altered TE and RNA DE were defined as described in Table 1.

Overall, we observed several similarities in the classes of genes that were induced at both the translational and transcriptional levels during early *C. albicans* biofilm formation. More specifically, gene classes associated with ribosomes/translation, oxidoreductase activity, response to drug, transporter activity, nucleic acid binding activity, and plasma membrane were highly represented in both sets (Fig. 4; Data set S2). Gene classes associated with structural molecule activity and budding/polarized growth showed a strong representation in the translationally vs transcriptionally induced gene sets (Fig. 4; Data set S2). Interestingly, however, there was no overlap between genes that showed increased transcription and those showing increased translation during early *C. albicans* biofilm formation, although a small fraction (4.9%) of genes with increased TE were transcriptionally downregulated (Fig. 5A; Data set S1), which included *PCL7* and *HOF1*, involved in cytokinesis and DNA damage response, as well as *PGA13*, a GPI-anchored cell wall protein involved in cell wall biosynthesis (Data set S1). Only two gene classes were in the top five most highly represented for the sets of genes that were translationally and transcriptionally downregulated during biofilm formation (plasma membrane and transporter activity) (Fig. 4; Data set S2), and only a single gene (the *ZRT3* membrane zinc transporter) was downregulated at both the transcriptional and translational levels (Fig. 5B; Data set S1), indicating very little overlap. Interestingly, however, 10.4% of genes with reduced TE showed increased RNA DE (Fig. 5B; Data set S1). These genes included two amino acid permeases (*HIP1* and *GAP6*), *FAS1*, encoding the β subunit of fatty acid synthase, *SDA1*, a putative nuclear protein involved in actin organization, and the *ALS1* α agglutinin-like adhesin. Our results suggest that while certain similar classes of genes are translationally and transcriptionally induced or downregulated, there is very little overlap, if any, among the specific genes that are regulated at the translational vs transcriptional level during early *C. albicans* biofilm formation.

## DISCUSSION

*C. albicans* biofilms promote immune evasion, show strong resistance to antifungals, and pose a serious threat to immunocompromised patients. While several previous studies have examined the transcriptional profile of early *C. albicans* biofilm formation (23, 26, 27), this study provides the first picture of the global translational response during this process. We observed that several gene classes were strongly induced at both the translational and transcriptional levels. These included gene classes associated with ribosomes/translation, plasma membrane, oxidoreductase activity, response to drugs, nucleic acid binding, and transporter activity. These findings suggest that early *C. albicans* biofilm formation is associated with increased protein synthesis and gene expression and requires the transport of key nutrients. Our results are also consistent with previous studies showing that genes important for intracellular transport, adhesion, and nucleic acid synthesis are upregulated at the transcriptional level early in *C. albicans* biofilm development (23, 26, 27). These results are expected, given the important roles that cellular adhesion and morphogenesis (which is associated with significant gene expression changes) play in *C. albicans* early biofilm formation (13, 16). However, other gene classes, such as site of polarized growth and cellular bud, are only upregulated at the translational level (Fig. 4; Data set S2), suggesting that *C. albicans* specifically uses translational mechanisms for inducing the expression of genes associated with these processes during early biofilm development. In contrast, other gene classes, such as helicase activity, translation regulator activity, and peroxisome are specifically upregulated at the transcriptional level (Fig. 4; Data set S2).

Our identification of specific genes that are translationally induced during early *C. albicans* biofilm formation provides new insights into the complexity of regulatory mechanisms that control this process. For example, several signaling components involved in promoting hyphal development showed increased TE during early *C. albicans* biofilm formation, which is consistent with the role of hyphal filaments in providing structure and architecture to developing biofilms (13, 16). However, at the same time, *NRG1*, a well-characterized strong transcriptional repressor of filament-specific genes and filamentation (50), also showed increased translation. Given that the *NRG1* transcript is typically downregulated under conditions that promote filamentation, increased *NRG1* TE may reflect a fine-tuning mechanism to very precisely control the level of filamentation within *C. albicans* biofilms. Interestingly, we also observed that Pop2, a key component of the *C. albicans* Ccr4/Pop2 mRNA deadenylase complex important for promoting mitochondrial stability, filamentation, and cell wall integrity (36), was also induced at the translational level during early biofilm formation. These findings suggest complex and multifaceted regulation, in which translational regulation induces a post-transcriptional mRNA stability mechanism, which, in turn, controls cell wall biogenesis and stability during early *C. albicans* biofilm formation. In addition, we observed that *CRZ2*, which is critical for yeast cell adherence and has previously been shown to be transcriptionally induced in *C. albicans* biofilms formed in Spider medium (25, 51), demonstrated strongly reduced TE in early-stage *C. albicans* biofilms. Similarly, the *ALS1* adhesin, which we and others have shown is transcriptionally induced during *C. albicans* biofilm formation (26, 27, 49, 52) (Data set S1), also showed significantly reduced translation. These findings suggest that genes important for the critical process of cell adhesion in biofilms may also be under the control of a complex translational fine-tuning mechanism.

It is important to note that while our analysis specifically provides information about changes in translational efficiency during *C. albicans* biofilm formation, protein degradation may also affect protein levels. For example, *NRG1*, which shows increased TE, is also known to be degraded at the protein level by a Sok1-mediated pathway during hyphal initiation (53). In addition, *UME6*, which has previously been shown to be controlled by a 5’ UTR-mediated translational efficiency mechanism, is also regulated at the level of protein stability by a Ptc2-Ssn3 CO_2_ signaling pathway (37, 53).

Overall, as expected, our RNA-seq analysis identified several genes that had previously been shown to be transcriptionally upregulated during *C. albicans* biofilm formation, including *ALS1* adhesin described above, *NCE103* carbonic anhydrase, as well as *MET3* and *MET14*, involved in sulfur assimilation (23, 26, 27, 49, 52). Most notably, however, we also observed strong transcriptional induction of genes important for protein synthesis and nucleolar functions, which highlights the need for rapid protein production during early *C. albicans* biofilm development.

While we observed both similarities and differences in the classes of genes that were translationally and transcriptionally upregulated during early *C. albicans* biofilm development, we observed no overlap among specific genes that were upregulated at the translational vs transcriptional levels (Fig. 5A; Data set S1). While several of the translationally induced genes (Table 2) have previously been shown to be transcriptionally induced when *C. albicans* cells formed biofilms under different growth conditions, others were previously demonstrated to be transcriptionally downregulated in biofilms (24, 25, 54), perhaps suggesting a similar trend. Previous studies also showed differences in specific genes that were transcriptionally induced or repressed when *C. albicans* forms biofilms under different conditions (e.g., rat catheter vs Spider medium) (24, 25). A similar pattern of very little overlap was also observed for genes that were downregulated at the translational vs transcriptional levels (Fig. 5B; Data set S1). Overall, these findings suggest that while certain common processes may be induced or downregulated during biofilm formation, *C. albicans* appears to use distinct translational and transcriptional mechanisms for regulating specific genes involved in these processes. Using ribosome profiling, we previously observed a similar finding for *C. albicans* genes that were induced at the translational vs transcriptional level in response to treatment with the mainline antifungal drug fluconazole (43). In addition, our previous analysis of the *C. albicans* global translational profile during morphogenesis revealed that many transcriptionally induced genes involved in virulence-related processes were significantly downregulated at the translational level (44). These previous findings, combined with our current analysis of the translational profile of early *C. albicans* biofilm development, suggest that *C. albicans* has evolved distinct translational and transcriptional mechanisms to mediate a variety of virulence-related processes. Given that certain genes appear to be exclusively regulated at the translational, but not transcriptional, level, these studies also highlight the importance of translational regulation for driving virulence and virulence-related processes in human fungal pathogens.

## MATERIALS AND METHODS

### *Candida albicans* strain and biofilm/planktonic growth conditions

A 25-mL overnight culture of *C. albicans* strain DK318 (55) was grown in YEPD (yeast extract peptone dextrose) at 30°C. Cells were harvested and washed in sterile DPBS (Dulbecco’s phosphate-buffered saline (GIBCO)). The cell pellet was then resuspended and diluted to 1 × 10^6^ cells/mL in pre-warmed RPMI 1640 medium (Sigma-Aldrich). Biofilms were formed in six 150-cm^2^ canted neck cell culture flasks (Corning) by adding 30 mL of pre-warmed RPMI 1640 medium containing 1 × 10^6^ cells/mL. The flasks were incubated at 37°C for 6 hours at 25 RPM. At the end of the incubation, the medium was carefully aspirated from the flasks, and the biofilm was washed three times with ice-cold sterile DPBS to remove non-adherent cells. Biofilm cells were then scraped off the flasks and collected by rapid filtration. Next, the pellet was resuspended in lysis buffer (1 x yeast polysome buffer (Illumina), 1% Triton X-100, (Sigma), 50 µg/mL GMPPNP (Sigma), 10 µg/mL Blasticidin S (InvivoGen)), snap-frozen in liquid nitrogen, and stored at −80°C until use. Planktonic cultures were grown by inoculating an equivalent number of cells from the same overnight culture used to initiate biofilm formation in 100 mL of pre-warmed RPMI 1640 medium. The culture was incubated at 37°C for 6 hours at 200 RPM. Cells were harvested by rapid filtration, and the pellet was washed three times with ice-cold sterile DPBS. The planktonic culture cell pellet was suspended in lysis buffer, snap-frozen, and stored at −80°C as described above. The experiments were performed using three independent biological replicates.

### Cell extract preparation and ribosome profiling

Cell lysates were prepared as described previously (44), except that samples were lysed by bead beating six times for 1 minute each with a 1-minute rest on ice between vortexes. Ribosome profiling was performed using an Illumina TrueSeq Ribo Profile (Yeast) Kit with several modifications as described previously (43, 44).

### Ribosome profiling and RNA-seq data analysis

Determination of read quality, trimming of adapters, Gene Ontology (GO) analysis, and identification of genes with altered TE and RNA DE in biofilm vs planktonic cells were carried out as described previously (44). Genes were defined as showing altered TE if transcripts per million (TPM) were >1 in at least two biological replicates for both biofilm and planktonic conditions and absolute fold-change on a log_2_ scale was ≥1 with a non-adjusted *P*-value of ≤0.05. We opted to use non-adjusted *p*-values for determining the translational efficiency in our differential translational efficiency analysis as our primary focus was on identifying potential changes in translational regulation (effect size) using a small set of samples (3), and we have previously observed an inherent variability in data generated from ribosome profiling experiments (46). We would like to highlight that our strategy results in reducing the Type II error at the cost of increasing Type I error. Genes with differential RNA DE were defined as those with TPM >1 in at least two biological replicates for both biofilm and planktonic conditions and absolute fold-change on a log_2_ scale was ≥1 with a FDR-adjusted *p*-value of ≤0.05.

## Data Availability

Raw and processed RNA-seq and Ribo-seq data from this study are available at the NCBI Gene Expression Omnibus (GEO; https://www.ncbi.nlm.nih.gov/geo/) database under accession number GSE274931. Custom scripts used to generate the manuscript data are available at Github: https://github.com/saketkc/2024_Albicans_Biofilm.

## SUPPLEMENTAL MATERIAL

Data S1 spectrum.02195-24-s0001.xlsx *C. albicans* biofilm Ribo-seq and RNA-seq data.

Data S2 spectrum.02195-24-s0002.xlsx *C. albicans* biofilm Ribo-seq and RNA-seq gene ontology (GO) data.

Supplemental material spectrum.02195-24-s0003.pdf Fig. S1 to S3.

ASM does not own the copyrights to Supplemental Material that may be linked to, or accessed through, an article. The authors have granted ASM a non-exclusive, world-wide license to publish the Supplemental Material files. Please contact the corresponding author directly for reuse.
