# Supplementary material for: Ribosome profiling reveals differences in global translational vs transcriptional gene expression changes during early Candida albicans biofilm formation
Source: Microbiol Spectr. 2025 Jan 28;13(3):e02195-24. doi: 10.1128/spectrum.02195-24 (PMC11878023; doi:10.1128/spectrum.02195-24)
Supplement: Supplemental material — Fig. S1 to S3. [file spectrum.02195-24-s0003.pdf]

SUPPLEMENTAL MATERIAL

Supplemental Figure Legends

Figure S1. Ribo-seq sample biological replicates show strong consistency. Correlation of TPM counts by scatter plots for all three Ribo-seq biological replicates under biofilm and planktonic conditions is shown.

Figure S2. RNA-seq sample biological replicates show strong consistency. Correlation of TPM counts by scatter plots for all three RNA-seq biological replicates under biofilm and planktonic conditions is shown.

Figure S3. Read coverage plot examples for additional selected *C. albicans* genes showing differential translational efficiency (TE) when cells were grown under biofilm vs. planktonic conditions. Normalized RNA-seq and Ribo-seq average read coverage across all replicates is shown for *CRZ2* and *RAM2* (A), which show reduced TE, as well as *DAP1* and *THI6* (B), which show increased TE.

Supplemental Datasets

Dataset S1. Translational efficiency (TE) and RNA differential gene expression (DE) data for *C. albicans* cells grown under biofilm vs. planktonic conditions. Gene annotations are from the *Candida* Genome Database (http://www.candidagenome.org).

Dataset S2. Gene Ontology (GO) data for *C. albicans* genes showing altered translational efficiency (TE) and RNA differential gene expression (DE) when cells were grown under biofilm vs. planktonic conditions.

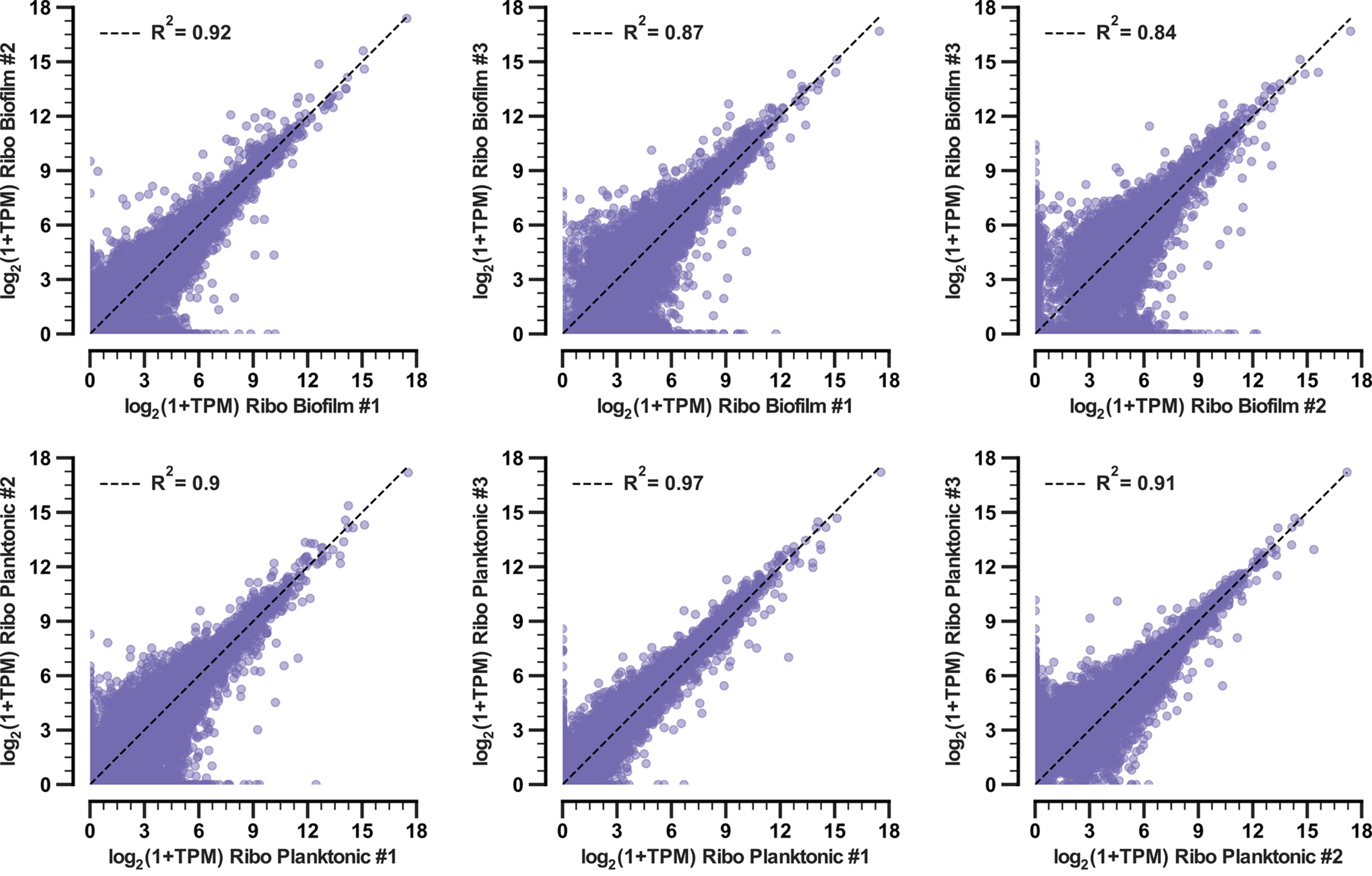

R^2 = 0.92
log2(1+TPM) Ribo Biofilm #2
log2(1+TPM) Ribo Biofilm #1
R^2 = 0.87
log2(1+TPM) Ribo Biofilm #3
log2(1+TPM) Ribo Biofilm #1
R^2 = 0.84
log2(1+TPM) Ribo Biofilm #3
log2(1+TPM) Ribo Biofilm #2
R^2 = 0.9
log2(1+TPM) Ribo Planktonic #2
log2(1+TPM) Ribo Planktonic #1
R^2 = 0.97
log2(1+TPM) Ribo Planktonic #3
log2(1+TPM) Ribo Planktonic #1
R^2 = 0.91
log2(1+TPM) Ribo Planktonic #3
log2(1+TPM) Ribo Planktonic #2

Figure S1

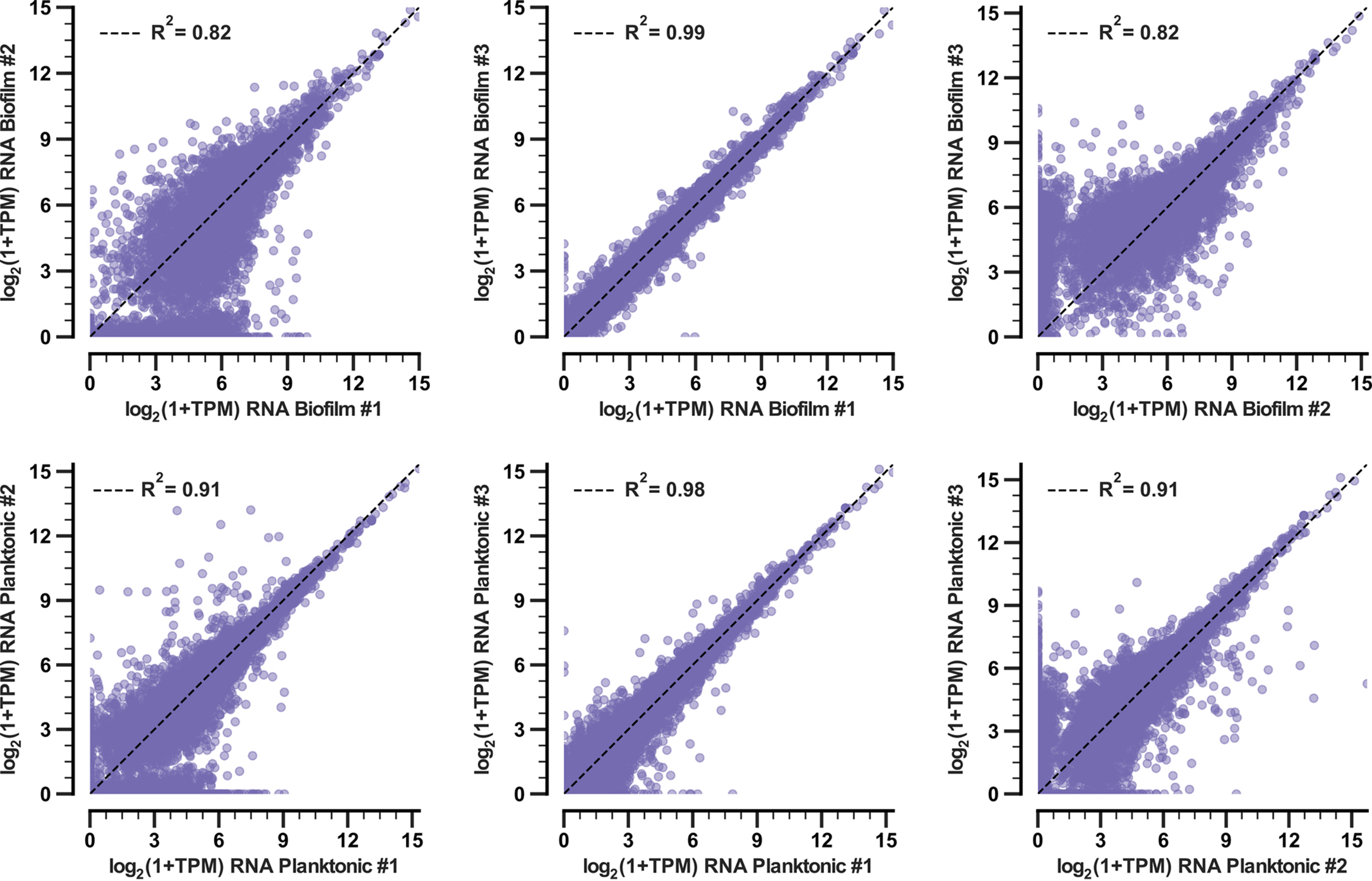
R^2 = 0.82
log2(1+TPM) RNA Biofilm #2
log2(1+TPM) RNA Biofilm #1
R^2 = 0.99
log2(1+TPM) RNA Biofilm #3
log2(1+TPM) RNA Biofilm #1
R^2 = 0.82
log2(1+TPM) RNA Biofilm #3
log2(1+TPM) RNA Biofilm #2
R^2 = 0.91
log2(1+TPM) RNA Planktonic #2
log2(1+TPM) RNA Planktonic #1
R^2 = 0.98
log2(1+TPM) RNA Planktonic #3
log2(1+TPM) RNA Planktonic #1
R^2 = 0.91
log2(1+TPM) RNA Planktonic #3
log2(1+TPM) RNA Planktonic #2

Figure S2

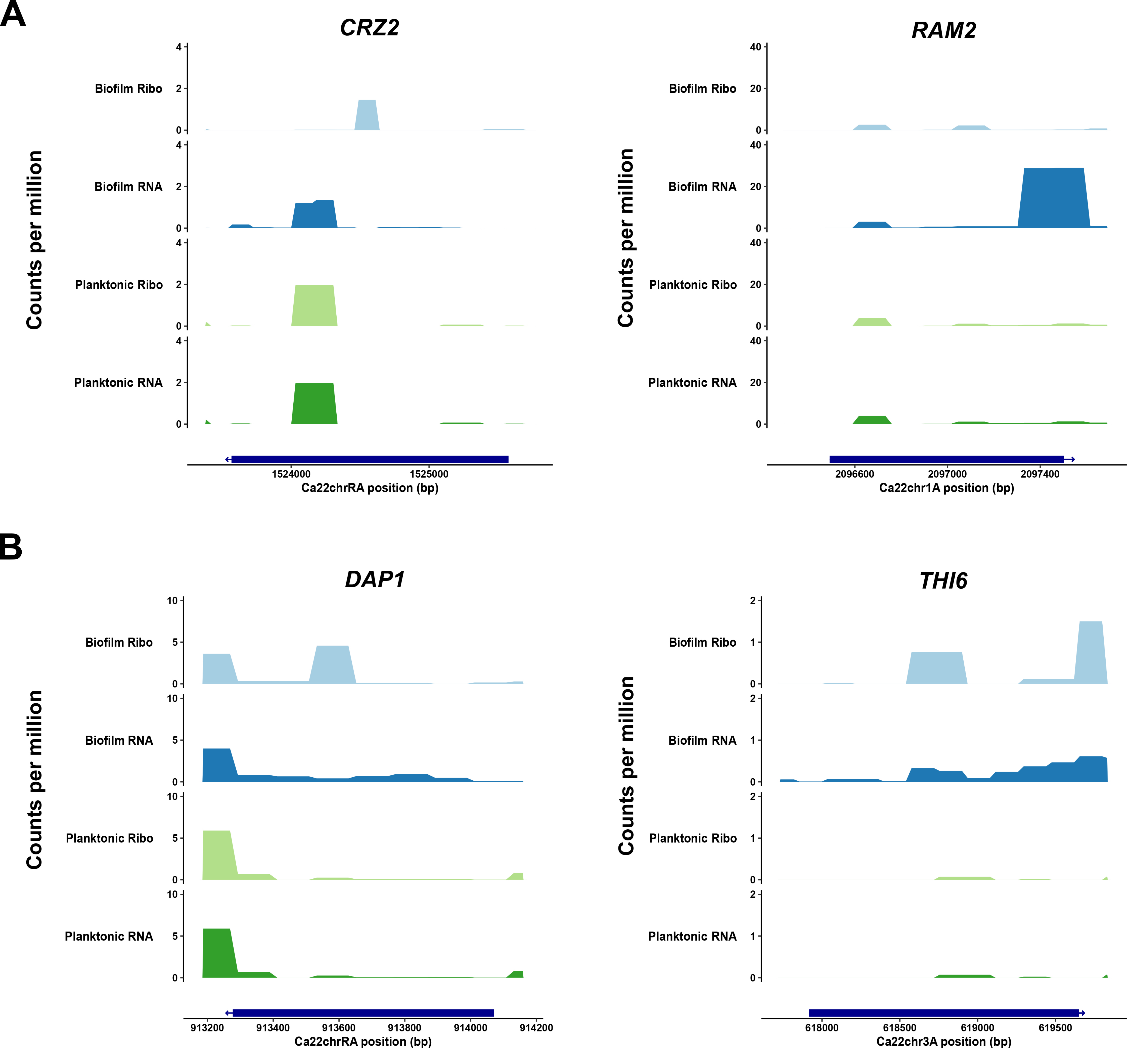
A
CRZ2
RAM2
Counts per million
Biofilm Ribo
Biofilm RNA
Planktonic Ribo
Planktonic RNA
1524000
1525000
Ca22chrRA position (bp)
2096600
2097000
2097400
Ca22chr1A position (bp)
B
DAP1
THI6
913200
913400
913600
913800
914000
914200
Ca22chrRA position (bp)
618000
618500
619000
619500
Ca22chr3A position (bp)

Figure S3
